# Barriers and facilitators to the implementation of osteoarthritis management programmes in primary or community care settings: a systematic review and qualitative framework synthesis protocol

**DOI:** 10.12688/hrbopenres.13377.1

**Published:** 2021-09-17

**Authors:** Joice Cunningham, Andrew M. Briggs, Elizabeth Cottrell, Frank Doyle, Krysia Dziedzic, Andrew Finney, Paul Murphy, Zoe Paskins, Eoin Sheridan, Laura Swaithes, Helen P. French

**Affiliations:** 1 School of Physiotherapy, Royal College of Surgeons in Ireland (RCSI) University of Medicine and Health Sciences, Dublin, Ireland; 2 Curtin School of Allied Health, Faculty of Health Sciences, Curtin University, Perth, Western Australia, Australia; 3 Impact Accelerator Unit, Versus Arthritis Primary Care Centre, School of Medicine, Keele University, Staffordshire, ST5 5BG, UK; 4 Department of Health Psychology, Royal College of Surgeons in Ireland, University of Medicine and Health Sciences, Dublin, Ireland; 5 RCSI Library, RCSI University of Medicine and Health Sciences, Dublin, Ireland; 6 Haywood Academic Rheumatology Centre, Midland Partnership NHS Foundation Trust, Stoke-on-Trent, ST6 7AG, UK

**Keywords:** barriers, facilitators, implementation, osteoarthritis management, primary care, community care, qualitative, framework synthesis

## Abstract

Despite consistent international guidelines for osteoarthritis (OA) management, evidence-based treatments are underutilised. OA management programmes (OAMPs) are being implemented internationally to address this evidence-practice gap. An OAMP is defined as a ‘model of evidence-based, non-surgical OA care that has been implemented in a real-world setting’. Our objective is to identify, synthesise and appraise qualitative research identifying anticipated or experienced micro (individual/behavioural), meso (organisational) or macro (context/system) level barriers or facilitators to the implementation of primary or community care-based OAMPs. Five electronic databases will be searched for papers published between 2010 and 2021. Qualitative or mixed-methods studies that include qualitative data on the anticipated or experienced barriers or facilitators to the implementation of primary or community care-based OAMPs, from the perspective of service users or service providers, will be included. The review will be reported using the PRISMA and ENTREQ guidelines. A data extraction form will be used to provide details of the included studies. Data will be analysed and identified barriers and facilitators will be mapped onto an appropriate implementation framework, such as the Theoretical Domains Framework. The appropriate JBI critical appraisal tools will be used to assess methodological quality, while the GRADE‐CERQual approach will be used to assess confidence in the findings. Translation of evidence-based guidelines into practice is challenging and reliant on the quality of implementation. By comparing and contrasting anticipated and experienced barriers, this review will determine the extent of congruence between the two, and provide valuable insights into the views and experiences of key stakeholders involved in the implementation of OAMPs. The mapping of identified barriers and facilitators to behaviour change theory will enhance the applicability and construct validity of our findings and will offer significant utility for future development and implementation of OAMPs.

**Registration:** This protocol was registered with PROSPERO (CRD42021255698) on 15/07/21.

## Introduction

Osteoarthritis (OA) is the most common joint disease worldwide and is one of the fastest growing causes of years lived with disability ^1^. OA is associated with an extremely high individual, economic and societal burden, frequently affecting multiple joint sites ^2^ and accompanied by comorbid physical and mental health conditions, increased mortality, and decreased participation and quality of life ^3, 4^. The World Health Organisation (WHO) estimates that by the year 2050, 130 million people worldwide will have OA and 40 million will be severely disabled by OA ^5^, representing a growing global public health concern ^6, 7^. Public health interventions are required to address the existing overuse of inappropriate and low-value care and provide equitable access to cost-effective, evidence-based management (high-value care) ^8^. Although numerous international clinical practice guidelines have been published endorsing exercise, education and weight management as first-line core treatments ^9– 12^, delivery and uptake of these treatments remains suboptimal ^13, 14^ and not consistently aligned with patient preferences ^15^.

Efforts to optimise evidence-based non-surgical treatments have led to the development and implementation of primary care-based OA management programmes (OAMPs) internationally ^16– 19^. In response to growing international development of OAMPs, the Osteoarthritis Research Society International (OARSI) OAMP Joint Effort Initiative (JEI) has been established. This is a collaboration of international researchers progressing the standardisation and harmonisation of the development and delivery of OAMPs ^20^. The OAMP JEI define an OAMP as a ‘model of evidence-based, non-surgical OA care that has been implemented in a real-world setting and includes the following four components: personalised OA care; delivered as a package of care with longitudinal reassessment and progression; comprising two or more elements of the core, guideline-recommended first line interventions (education, exercise and weight loss); with optional adjunct treatments as required (e.g. assistive devices and psychosocial support)” ^21^. OAMPs focussing on education and exercise for patients with knee, hip and hand OA have shown positive effects on patient outcomes including pain, physical function, health-related quality of life and self-efficacy in developed countries ^19, 22, 23^. Reduced sick leave and analgesia use ^19^, decreased willingness for surgery ^24^ and delayed knee or hip replacement surgery ^25, 26^ have also been reported.

However, the potential benefits of primary care-based OAMPs may be constrained by barriers to their effective and sustained implementation. These may occur at the micro (individual behavioural, including clinicians and consumers), meso (organisational) or macro (context and system) levels ^27^. Successful translation of evidence into clinical practice can be challenging and requires a comprehensive and targeted micro-, meso- and macro-level approach adapted to the specific setting and relevant stakeholders ^28, 29^. An understanding of anticipated or experienced barriers and facilitators to implementation is vital to realise the full value of OAMPs. Furthermore, evidence on the factors that facilitate this process could improve recruitment to, and uptake of, future OAMPs. Barrier themes identified in a previous systematic review exploring clinicians’ perceived barriers and enablers to management of OA in primary care included: clinician beliefs, knowledge gaps, communication, behaviour change skills, dissonant patient expectations and co-morbidities which increase complexity of care ^30^, while no enabler themes were identified. A recent systematic review also identified factors influencing implementation of evidence-based guidelines in primary care settings including: research and ‘real-world’ disconnects, views of patients and primary care clinicians and engagement of whole primary care practice settings in the implementation process ^31^. However, the anticipated and experienced barriers have yet to be combined and subsequently compared and contrasted. Hence, this review will explore whether, for example, clinicians’ concerns or fears are corroborated by experienced barriers. Furthermore, unlike earlier reviews, this review will be theory-informed, barriers and facilitators will be reviewed through a micro-meso-macro level lens and will be linked to evidence-based behaviour change theory. Our a priori framework, the Theoretical Domains Framework (TDF), is a comprehensive evidence-based implementation framework developed for systematically identifying and assessing barriers to change interventions ^32, 33^. By mapping the data to the TDF, this review will add to the current literature and will inform future intervention design and allow for tailored implementation solutions.

### Study aim

This review aims to identify, synthesise and appraise the qualitative evidence on barriers and facilitators to the implementation of primary or community care-based OAMPs. 

### Objectives

- To describe the anticipated or experienced micro-, meso- or macro-level barriers or facilitators to the implementation of primary or community care-based OAMPs from the perspectives of patients, carers, healthcare professionals and other relevant stakeholders across these levels.
- To compare and contrast anticipated and experienced facilitators.
- To map the identified barriers and facilitators to the TDF domains.

## Methods

### Guidelines and registration

The Preferred Reporting Items for Systematic review and Meta-Analysis (PRISMA) guidelines ^34^ and the Enhancing Transparency in Reporting the Synthesis of Qualitative Research (ENTREQ) guidelines ^35^ will be used to guide the conduct of this review. The review protocol was registered in the International Prospective Register of Systematic Reviews (PROSPERO), ID: CRD42021255698, on 15/07/21.

### Search strategy

To identify eligible studies, we will search electronic databases including Ovid MEDLINE, EMBASE, CINAHLvia EBSCOhost, PsychINFO, and Web of Science from 2010 onwards to present to align with publication dates of recent guidelines. A combination of Mesh terms and keywords will be developed in conjunction with a medical librarian. The search strategy will include terms related to ‘osteoarthritis’, ‘implementation’, ‘management programme’, ‘primary and community care’ and ‘qualitative design’ with Boolean operator “and”. An academic librarian will be consulted regarding the appropriate Medical Subject Heading (MeSH) terms used for each electronic database. This will be initially tested in Ovid MEDLINE and adapted for all other databases. The search strategy will be restricted to English language articles published from 2010 onwards. A sample search strategy for Ovid MEDLINE is available (see extended data) ^36^. The OAMP JEI discussion group established by OARSI will be contacted to identify other relevant research. This platform has been deemed suitable given its international reach.

### Article selection

Articles retrieved will be exported to Endnote X9 (Thomson Reuters [Scientific] LLC, Philadelphia, PA, USA) with duplicate entries removed. Titles and abstracts will be screened independently by two review authors (JC and AF or ES), according to the inclusion criteria. Any disagreements will be resolved through a third arbitrator (HPF). The same process will be employed for potentially eligible full-text publications. Finally, we will search for additional studies using the reference lists of identified articles. This will involve forward and backward citation checking of all identified systematic reviews and all articles eligible for inclusion will be conducted. 

### Eligibility criteria

The SPIDER (Sample, Phenomenon of Interest, Design, Evaluation, Research type) framework ^37^ will be used to identify eligibility criteria for study inclusion [ Table 1].

**Table 1.** The SPIDER framework.

	Inclusion criteria
***Sample***	Service users or providers involved in the process of implementing an OAMP. Service users: people with OA (≥18 years old). Service providers include: • Primary care clinicians such as GPs or other primary care clinicians. • Community care providers such as personal trainers, gym instructors, strength and conditioning coaches. • Individuals involved at an organisational or system level such as health service managers.
***Phenomenon of*** ***interest***	Micro-, meso-, or macro-level anticipated or experienced barriers or facilitators to implementing primary or community care-based OAMPs.
***Design***	Original qualitative research design, or studies in which primary qualitative data can be extracted (e.g., mixed methods research).
***Evaluation***	Studies must provide qualitative data which represent the perspectives of service users or providers.
***Research*** ***method***	Research published from 2010 onwards to align with publication dates of recent guidelines. Full-text articles OAMPs must be in line with the OARSI definition as detailed above. OAMPs must be primarily based in the primary or community care setting.

### Sample

The population of interest will include individuals involved in the implementation of an OAMP, namely service users (individuals receiving OA care and/or their carers, in the form of an OAMP) and service providers (individuals involved in providing primary or community care or other relevant stakeholders) as described in an included study. For individuals with OA, diagnosis may be based on radiographic criteria, clinical features or combination criteria ^38^, and will be determined by the original research articles. Primary care clinicians may include general practitioners (GPs), nurses, physiotherapists, occupational therapists, dieticians, podiatrists etc. Community care service providers may include health workers, lay and professional, formal and informal, paid and unpaid, involved in the implementation of an OAMP, for example personal trainers, gym instructors, strength and conditioning coaches, community leaders etc. Other relevant stakeholders may include individuals involved at an organisational or system level such as health service managers, policy makers, funders/insurers or service commissioners. 

### Phenomenon of interest

The phenomenon of interest includes anticipated or experienced barriers or facilitators to implementing community or primary care-based OAMPs. At least one service user or provider-related barrier or facilitator theme must be described in a primary study to be included in this review. Experiences of OA not related to an OAMP will not be eligible for inclusion, but the explicit use of the term ‘OAMP’ is not required for inclusion. For the purpose of this review, an OAMP will be defined as per the OARSI OAMP JEI as a ‘model of evidence-based, non-surgical OA care that has been implemented in a real-world setting’ ^20^. It should comprise the following four components: ‘personalised OA care; provided as a package of care with longitudinal reassessment and progression; comprising two or more components of the core, non-surgical, non-pharmacological interventions (education, self-management support or exercise) and optional evidence-based adjunctive treatments as required (e.g., weight loss interventions, where appropriate, psychological support, review of analgesics and prescription of assistive devices)’ ^20^. The setting for initiation and delivery of the OAMP must be in primary or community care, but can include pathways for referring patients to secondary care. OAMPs delivered in secondary care or other specialist ambulatory settings will be excluded. We will adopt the World Health Organisation (WHO) definitions of primary and community care ^39, 40^. The OAMP may be implemented at a small-scale (e.g., local pilot or feasibility studies) or large-scale level (e.g. large-scale implementation studies).

### Design and evaluation

All identified full-text original research that provide qualitative data on anticipated or experienced barriers or facilitators to the implementation of primary or community care-based OAMPs will be included. We will consider primary studies using focus groups or one-to-one, in-depth or semi-structured interviews, explicitly reporting one or more barrier or facilitator. Surveys that provide qualitative data or studies of mixed methods design, where qualitative data can be extracted separately, will be included. Excluded study designs include stand-alone quantitative research, individual case reports (<10 participants), systematic or narrative reviews, conference proceedings and opinion/narrative/discussion and editorial articles.

### Research type

Included studies must be published from 2010 onwards to align with publication dates of recent guidelines. In the case of abstracts or protocols being retrieved, attempts will be made to access full-texts by contacting the authors.

### Data extraction and analysis

Two review authors (JC and HPF or ES) will extract descriptive study characteristics independently using a structured data extraction form. Detailed data and contextual information (including author, title, year of publication, country of origin, research aims, methodology, sample and setting, data collection methods and analysis, theoretical framework used, results and conclusions) will be extracted.

### Quality appraisal of the included studies

Methodological quality assessment of included studies will be performed using the appropriate Joanna Briggs Institute Critical Appraisal tool for qualitative and quantitative research ^41^. This will be conducted by two independent review authors (JC and AB) with a third review author (FD) available should any disagreements arise.

### Evidence synthesis

Findings will be synthesised using a framework synthesis, which is a review method that combines inductive and deductive approaches to synthesise empirical findings from qualitative research studies ^42^. Extracted data labelled ‘results’ from included studies will be imported into NVIVO version 12 (QRS International, Cambridge, MA, USA). A two-stage analytical approach will be employed incorporating 1) an inductive line-by-line coding of the extracted results to identify emergent themes, followed by 2) a deductive exercise to map identified codes to an a priori framework. During analysis we will adopt a micro-meso-macro level lens to ensure individual, organisational and broader factors are considered. Following extensive familiarisation with the data, two independent review authors (JC and HPF) will code the data. A third review author (LS) will inductively code a sample of transcripts to explore shared meanings and interpretations. The consistency of coding will be explored to facilitate the coding framework development. Codes with similar meanings will be grouped together to form broader themes, whilst checking for confirmatory or challenging evidence. At this stage, a final coding will be agreed through consensus discussion of codes and themes among a study panel of approximately six individuals (including review authors and members of a patient advisory panel). The coding framework will then be systematically applied to the whole dataset. 

Following this, the second stage of analysis will involve using a framework. Our a priori framework, the TDF, provides a robust theoretical basis for implementation studies, as it is one of the few frameworks linked to a comprehensive method for intervention design ^43, 44^. It allows researchers to capture the potentially broad range of potential determinants of implementation, which are relevant to behaviour change ^45– 47^. The refined version of the TDF consists of 14 domains ^48^ and 84 constructs relating to behaviour change theory ^49– 51^. Identified barrier and facilitator themes will be mapped according to the TDF: (1) knowledge, (2) skills, (3) social influences, (4) memory, attention and decision processes, (5) behavioural regulation, (6) professional/social role and identity, (7) beliefs about capabilities, (8) belief about consequences, (9) optimism, (10) intentions, (11) goals, (12) emotion, (13) environmental context and resources and (14) reinforcement. A coding manual with theoretical and working definitions and component constructs of each of the 14 domains will be used to operationalise the TDF and facilitate coding consistency ^43^. The framework will be adapted to accommodate any themes that cannot be mapped to a TDF domain. This mapping exercise will initially be carried out by two review authors (JC and FD), and then verified through detailed discussion with two other review authors (ZP and LS). This process will involve the examination of each data extract (exemplifying each theme), and exploring how these extracts fit (or did not fit) within the parameters of each theoretical domain. All codes will then be reviewed and themes will be discussed within the study panel to ensure consistency and ‘fit’ to the framework. A preliminary synthesis will be achieved using tabulation of studies, organising the studies into groups e.g. relating to anticipated and experienced barriers, and exploring relationships between studies and between groups e.g. comparing and contrasting anticipated and experienced barriers and identify areas of dissonance/concordance. A final coding framework will be agreed through consensus discussion of the themes and interpretations between the study panel. This method, commonly used in health research and TDF analyses, allows for exploration of unanticipated factors associated with implementation ^52^. The GRADE CERQual approach will be used to summarise confidence in the evidence ^53^, where each review finding will be assessed under four components: (1) methodological limitations, (2) coherence, (3) adequacy of data, and (4) relevance. This will be carried out independently by two review authors (JC and LS). A third review author will resolve any disagreements (FD). Following this process, an overall assessment of confidence in each finding will be made, and categorised as High, Moderate, Low or Very Low confidence ^54^.

### Dissemination of findings

On completion of the analysis, this review will be submitted for publication in a high-ranking international peer-reviewed journal. It will be shared with the Implementation subgroup of OARSI’s Joint Effort Initiative and the JEI website resources for OARSI.

### Study status

At the time of publication of this protocol, database searches have been completed and study selection is underway. Completion of the review is expected by March 2022.

## Discussion

Musculoskeletal health conditions such as OA impart a substantial individual, socioeconomic and societal burden worldwide ^55^, particularly in the context of increasing life expectancy and obesity. Despite this, OA remains under-diagnosed and under-treated ^56^, when compared to non-musculoskeletal chronic diseases ^8^. There is an urgent need to address the shortcomings in the management of OA, given that OA commonly co-exists with other conditions such as hypertension, cardiovascular disease and diabetes ^57^, and worsens the morbidity and mortality associated with these chronic diseases ^8^. While the majority of research on musculoskeletal disorders to date has been conducted in high-income settings, limited data suggest that the prevalence of arthritis may be equivalent or higher in lower-middle-income countries ^58^. The development of OAMPs internationally remains in its infancy, and they have been for the most part implemented at a relatively small-scale ^20^ and typically in high income nations. Therefore, a comprehensive understanding of the barriers and facilitators to implementation of OAMPs represents an important area of implementation research and informing scalability of OAMPs. By mapping the barriers and facilitators to the TDF, this review will provide a theoretical pathway to inform intervention and guide service designers and implementers in identifying and overcoming likely micro-, meso- or macro-level barriers to sustainable service delivery. This theory-based approach will enhance adoption of future OAMPs into community and primary care settings and narrow the evidence-practice gap by informing future research in developing targeted efficacious management programmes and implementation strategies in high, middle and low-income countries.

## Conclusion

The findings of this qualitative synthesis will provide valuable information on the anticipated or experienced barriers or facilitators to the implementation of primary or community care-based OAMPs. By mapping the identified barriers and facilitators to behaviour change theory, this review will help to reduce the evidence-practice gap in future development and implementation of OAMPs.

## Data availability

### Underlying data

No underlying data are associated with this article.

### Extended data

Open Science Framework: Barriers and facilitators to the implementation of osteoarthritis management programmes in primary or community care settings; a qualitative framework synthesis protocol. https://doi.org/10.17605/OSF.IO/YGTCN ^36^. 

This project contains the following extended data:

- Additional file 1 Ovid MEDLINE Preliminary Search Strategy.pdf

### Reporting guidelines

Open Science Framework: PRISMA-P checklist ^59^ for ‘Barriers and facilitators to the implementation of osteoarthritis management programmes in primary or community care settings; a qualitative framework synthesis protocol’. https://doi.org/10.17605/OSF.IO/YGTCN ^36^.

Data are available under the terms of the Creative Commons Attribution 4.0 International license (CC-BY 4.0).
