# Supplementary material for: Barriers and facilitators to the implementation of osteoarthritis management programmes in primary or community care settings: a systematic review and qualitative framework synthesis protocol
Source: HRB Open Res. 2021 Sep 17;4:102. [Version 1] doi: 10.12688/hrbopenres.13377.1 (PMC8543168; doi:10.12688/hrbopenres.13377.1)
Supplement: Peer review history

# Peer review: Reviewer response for version 1

**DOI:** 10.21956/hrbopenres.14571.r30322

**Published:** 2021-10-26

**Authors:** Joost Dekker

**Affiliations:** 1 Department of Rehabilitation Medicine, Amsterdam UMC, Location VUmc, Amsterdam, The Netherlands

This is the protocol for a systematic review of barriers and facilitators to the implementation of osteoarthritis management programs in primary or community care settings as reported in qualitative studies. It is a rather straightforward protocol. My most important comment concerns the relevance of this review. Two systematic reviews on the same topic haven been published. The authors’ state two reasons for doing another review.

 First, the authors aim to map the identified barriers and facilitators to behavior change theory and at the micro-, meso- or macro-level. They expect this to enhance the applicability and construct validity of their findings. This is all done a posteriori (post hoc). I am not so sure that a post hoc categorization of barriers/facilitators adds much to what we know or to the applicability of the findings. I am not against it, but I just do not think that at this stage this is very helpful. The findings in previous systematic reviews can be mapped to behavior change theory and at the micro-, meso- or macro-level.

 Second, they aim to compare anticipated and experienced barriers, and they expect this to provide valuable insights. I am not sure that anticipated and experienced barriers are different: I would expect that anticipated barriers are based on actual experiences.

 So, I am not against this review, but I think that our efforts should now focus on theory- and evidence-driven efforts to improve implementation of osteoarthritis management programs. I doubt that the review will add much to what we already know.

 I have only two comment on the methods.

- An osteoarthritis management program is defined as a ‘model of evidence-based, non-surgical OA care ….’. What is the meaning of ‘evidence-based’ ? Does this concern evidence for the effectiveness of the entire program, or for the components of the program? How much evidence is needed to include a program/study, or conversely: how little evidence results in exclusion of the program/study?
- A definition of micro-, meso- and macro-level is needed.

Is the study design appropriate for the research question?

Yes

Is the rationale for, and objectives of, the study clearly described?

Partly

Are sufficient details of the methods provided to allow replication by others?

Yes

Are the datasets clearly presented in a useable and accessible format?

Not applicable

Reviewer Expertise:

Rehabilitation, osteoarthritis

I confirm that I have read this submission and believe that I have an appropriate level of expertise to confirm that it is of an acceptable scientific standard.

# Peer review: Reviewer response for version 1

**DOI:** 10.21956/hrbopenres.14571.r30378

**Published:** 2021-10-21

**Authors:** Allison M. Ezzat

**Affiliations:** 1 La Trobe Sport and Exercise Medicine Research Centre, School of Allied Health, Human Services and Sport, La Trobe University, Bundoora, Vic, Australia

Thank you for the opportunity to review this study protocol on barriers and facilitators to implementation of osteoarthritis management. This is a well written protocol describing an novel systematic review that will use a theory driven approach to improve the implementation of future OA community programs. I only have a few suggestions:

1. It is not clear if the authors will include community-based OA programs that are implemented virtually or online? Please clarify with study inclusion criteria.
2. Would authors consider mapping the results of real and anticipated barriers and enablers to the Behaviour Change Wheel intervention functions to illustrate concrete examples of evidence-based behaviour change strategies to target these found barriers and enablers? This would provide potential solutions to these key problems. 

Is the study design appropriate for the research question?

Yes

Is the rationale for, and objectives of, the study clearly described?

Yes

Are sufficient details of the methods provided to allow replication by others?

Yes

Are the datasets clearly presented in a useable and accessible format?

Yes

Reviewer Expertise:

Implementation science with focus on knee injury prevention and knee osteoarthritis management

I confirm that I have read this submission and believe that I have an appropriate level of expertise to confirm that it is of an acceptable scientific standard.

# Peer review: Reviewer response for version 1

**DOI:** 10.21956/hrbopenres.14571.r30379

**Published:** 2021-10-11

**Authors:** Thèrèse Jönsson

**Affiliations:** 1 Department of Health Sciences, Sport Sciences, Faculty of Medicine, Lund University, Lund, Sweden

**Overview:**

 This is a study protocol describing a systematic review and qualitative framework synthesis protocol for barriers and facilitators implementation of OA management programmes. This is a very important study to do. We know so much about the effects of OA management programs, but they are still not used in the “real world”.

**Comments:**

**1. Title:**

 The title is good and clear.

**2. Abstract:**

 The abstract is clear, but will the review include all OA management programs (shoulder, back, hip, knee, hand, foot etc) or only knee and hip? Please describe that both in the abstract and background.

**3. Keywords:**

 Maybe you should add osteoarthritis and hip, knee depending on what you will evaluate?

**4. Introduction and aim:**

 In the background you describe OA management programs internationally, I think you should add BOA with more than 150 000 patients included all over Sweden (a huge and successful implementation project):

1. Better Management of Patients with Osteoarthritis: Development and Nationwide Implementation of an Evidence-Based Supported Osteoarthritis Self Management Programme Carina A. Thorstensson, Göran Garellick, Hans Rystedt & Leif E. Dahlberg ^1^.
2. The Better Management of Patients with Osteoarthritis Program: Outcomes after evidence-based education and exercise delivered nationwide in Sweden. Therese Jönsson, Frida Eek, Andrea Dell’Isola, Leif E. Dahlberg, Eva Ekvall Hansson ^2^ (my article).

Otherwise, I think the background is good and clear.

**5. Materials and methods:**

 The method is good and clear.

**6. Discussion:**

 The discussion is good, clear, and relevant for the study.

**7. Conclusion:**

 The conclusion is good and clear.

Is the study design appropriate for the research question?

Yes

Is the rationale for, and objectives of, the study clearly described?

Yes

Are sufficient details of the methods provided to allow replication by others?

Yes

Are the datasets clearly presented in a useable and accessible format?

Yes

Reviewer Expertise:

Osteoarthritis, physical therapy, first-line treatment for osteoarthritis

I confirm that I have read this submission and believe that I have an appropriate level of expertise to confirm that it is of an acceptable scientific standard.

## References

[1] : Better Management of Patients with Osteoarthritis: Development and Nationwide Implementation of an Evidence-Based Supported Osteoarthritis Self-Management Programme. *Musculoskeletal Care* .2015;13(2) : 10.1002/msc.1085 67-75 10.1002/msc.1085 25345913

[2] : The Better Management of Patients with Osteoarthritis Program: Outcomes after evidence-based education and exercise delivered nationwide in Sweden. *PLoS One* .2019;14(9) : 10.1371/journal.pone.0222657 e0222657 10.1371/journal.pone.0222657 31536554PMC6752869
